# End-Expiratory Occlusion Test and Tidal Volume Challenge Test for Evaluating Fluid Responsiveness in Severe Traumatic Brain Injury, Septic Shock, and Acute Respiratory Distress Syndrome

**DOI:** 10.7759/cureus.80581

**Published:** 2025-03-14

**Authors:** Panu Boontoterm, Siraruj Sakoolnamarka, Peera Naklaor, Karanarak Urasyanandana

**Affiliations:** 1 Neurological Surgery, Phramongkutklao Hospital, Bangkok, THA

**Keywords:** ards (acute respiratory distress syndrome), end expiratory occlusion test, fluid responsiveness, severe traumatic brain injury, tidal volume challenge test

## Abstract

Introduction: Fluid management in critically ill patients, particularly those with severe traumatic brain injury (TBI), septic shock, and acute respiratory distress syndrome (ARDS), presents a complex and multifaceted challenge. Dynamic tests such as the end-expiratory occlusion (EEO) test and tidal volume challenge (TVC) test are commonly used to assess fluid responsiveness, providing valuable insights into cardiovascular responses to changes in volume status. However, due to the unique risks and complications associated with these conditions, there is an increasing need to explore and evaluate alternative methods for predicting fluid responsiveness more safely and accurately in these critically ill patients.

Methods: This study presents a prospective investigation conducted on patients with severe TBI, septic shock, and ARDS. Before administering a 100 mL colloid bolus, both the EEO and TVC tests were performed. Initial measurements of cardiac output (CO), cardiac index (CI), and pulse pressure variation (PPV) were recorded, followed by subsequent measurements after each test to assess the fluid responsiveness and cardiovascular changes in these critically ill patients.

Results: Among the 180 participants, a more than 5% increase in CI during the EEO test was indicative of fluid responsiveness. Similarly, a 3.5% absolute increase in PPV during the TVC test suggested fluid responsiveness. The interrater reliability for the EEO test was observed to be 0.915, indicating strong agreement between raters, while for PPV, it was 0.637, reflecting moderate agreement. These values suggest that the EEO test shows a high degree of consistency between different evaluators, whereas the PPV measurement demonstrates a more moderate level of reliability.

Conclusion: In patients with severe TBI, septic shock, and ARDS who are receiving low tidal volume (VT) ventilation, both the EEO test for 15 seconds and the TVC method can be used to assess fluid responsiveness. However, it is important to note that the EEO test demonstrates greater reliability in this context.

## Introduction

Fluid management in severe traumatic brain injury, septic shock, and acute respiratory distress syndrome

Fluid management in critically ill patients, particularly those with severe traumatic brain injury (TBI), septic shock, and acute respiratory distress syndrome (ARDS), presents a complex challenge. Accurate assessment of fluid responsiveness is essential to optimize hemodynamic status, enhance tissue perfusion, and prevent complications such as fluid overload or inadequate resuscitation [1,2]. In this patient population, both the end-expiratory occlusion (EEO) test and the tidal volume challenge (TVC) test are dynamic tools used to evaluate fluid responsiveness. These tests offer valuable insights into the cardiovascular response to changes in volume status, but their application must be carefully tailored to the unique risks and complications associated with severe TBI, septic shock, and ARDS [3-15].

End-expiratory occlusion test

The EEO test assesses fluid responsiveness by temporarily occluding the airway at the end of exhalation. This maneuver decreases intrathoracic pressure, which in turn increases venous return and stroke volume or cardiac output (CO). A significant increase in stroke volume after the release of the occlusion suggests that the patient is fluid-responsive and may benefit from additional fluid administration.

The EEO test has several advantages, particularly in patients with severe TBI, septic shock, and ARDS. It is non-invasive and can be performed at the bedside, making it highly useful in resource-limited settings or emergency situations. The EEO test dynamically measures the hemodynamic response in real time, providing more reliable insights than static parameters like central venous pressure (CVP). Furthermore, since patients with severe TBI, septic shock, and ARDS are typically mechanically ventilated, the EEO test can be easily synchronized with the ventilator to avoid interfering with the respiratory cycle.

Intracranial Pressure Concerns

The safety of the EEO test in patients with traumatic brain injury: The safety of the EEO test in patients with TBI is a critical consideration, especially due to the potential for increased intracranial pressure (ICP) during the test. TBI often leads to cerebral edema and impaired autoregulation of cerebral blood flow, making ICP management a vital component of patient care.

Potential Risks

Increased intrathoracic pressure and ICP concerns: In patients with severe TBI, septic shock, and ARDS, it is important to address several considerations and risks, particularly those related to ICP. During the EEO test, increasing intrathoracic pressure may raise ICP, potentially exacerbating neurological damage [8,15]. Therefore, continuous monitoring of ICP during the test is essential. If any deterioration in ICP is observed, the test should be immediately stopped to prevent further harm. The temporary occlusion of the airway at the end of exhalation increases intrathoracic pressure, which reduces venous return to the heart, transiently elevating CVP and potentially raising ICP. In TBI patients, this rise in ICP can compromise cerebral perfusion pressure (CPP), increasing the risk of secondary brain injury.

Cerebral perfusion and autoregulation: In TBI patients, the brain’s ability to autoregulate blood flow may be impaired. Therefore, changes in systemic blood pressure such as those caused by fluid shifts during the EEO test can have a more significant impact on cerebral perfusion. This could result in inadequate blood flow to the brain and further neurological compromise.

Safety Considerations

ICP monitoring: Continuous and careful monitoring of ICP is essential when performing the EEO test in TBI patients. Any increase in ICP should prompt immediate discontinuation of the test. Maintaining CPP within safe limits is crucial. Invasive monitoring tools, such as ICP devices and continuous hemodynamic assessments, should be used to mitigate potential adverse effects.

Patient Selection

The EEO test may be more appropriate for TBI patients with stable ICP who are not at risk for significant fluctuations during the procedure. However, in patients with severe TBI and unstable ICP, the test should either be avoided or used with extreme caution.

Fluid Balance and Acute Respiratory Distress Syndrome

In patients with ARDS, fluid overload can worsen pulmonary edema, impair gas exchange, and complicate respiratory management. The EEO test can help prevent unnecessary fluid administration by assessing fluid responsiveness and determining whether further volume loading would be beneficial or potentially harmful in these patients.

Fluid Responsiveness Assessment

The EEO test’s ability to assess fluid responsiveness can be valuable, particularly for patients with complex conditions such as TBI and septic shock. However, in TBI patients, decisions regarding additional fluid administration should be made carefully, as fluid overload could increase ICP and worsen outcomes. Therefore, while the EEO test can help avoid unnecessary fluid administration, its use must be carefully balanced to minimize risks.

The existing literature on the use of the EEO test in TBI patients is limited, and more research is needed to specifically address its safety in this population. Most studies evaluating the EEO test focus on general fluid responsiveness in mechanically ventilated patients or those with sepsis, rather than specifically in TBI cases. However, similar tests, such as passive leg raises and fluid challenges, have been studied in TBI patients, with some findings showing that increases in intrathoracic pressure can significantly impact ICP and cerebral perfusion. These studies highlight the need for caution when applying dynamic fluid responsiveness tests to this vulnerable patient population.

While the EEO test can be a useful tool for assessing fluid responsiveness in critically ill patients, its use in TBI patients with elevated ICP must be approached with caution. Close monitoring of ICP, careful patient selection, and thoughtful consideration of the test’s safety in this group are crucial to minimizing risks and ensuring the best possible outcomes.

Septic shock

*Fluid Resuscitation in Septic Shock and the Role of the End-Expiratory Occlusion* *Test*

Fluid resuscitation is a cornerstone in the management of septic shock, as it helps restore intravascular volume, improve tissue perfusion, and stabilize blood pressure. In septic shock, the body’s ability to maintain adequate blood flow is compromised due to widespread vasodilation and increased capillary permeability. Timely and appropriate fluid administration is therefore crucial to reverse shock and prevent organ failure.

The EEO test can help identify patients who are likely to benefit from additional fluids by assessing their fluid responsiveness. However, clinicians must be cautious of the potential for fluid overload, particularly in ARDS patients, as this can exacerbate respiratory distress and worsen pulmonary function [7].

Septic shock and acute respiratory distress syndrome

Approximately 10% of critically ill patients admitted to the intensive care unit (ICU) are affected by septic shock and ARDS [1]. Many of these patients present with an oxygenation ratio (P/F ratio) below 150 mmHg. The American Thoracic Society recommends lung-protective ventilation as a cornerstone of the treatment protocol. The primary goal of lung-protective ventilation is to minimize lung injury while maximizing oxygen exchange. This involves optimizing positive end-expiratory pressure (PEEP) settings and maintaining low tidal volumes (VTs) (6-8 mL/kg) to control driving pressures, ensuring they remain below 15 mmHg [2].

Acute respiratory distress syndrome

Approximately 40% of ARDS patients experience circulatory shock, requiring careful management of fluids [3]. While fluid resuscitation is essential, excessive fluid administration can lead to pulmonary edema, impairing oxygen exchange, and underscoring the need for precise fluid monitoring. Various techniques are used to assess fluid responsiveness in patients with circulatory shock, including pulse pressure variation (PPV), passive leg raising tests, and the inferior vena cava (IVC) distensibility index [4-6]. However, these methods have limitations, particularly in patients positioned prone and receiving low VT ventilation, where changes in intrathoracic pressure may interfere with accurate readings. Emerging techniques, such as the TVC, EEO, and mini-fluid challenge tests, are showing promise in overcoming these challenges, offering more reliable fluid assessment in these complex scenarios [6-9,16].

The EEO test can help identify patients who are likely to benefit from additional fluids by assessing their fluid responsiveness. However, clinicians must be cautious of the potential for fluid overload, especially in ARDS patients, as this can further impair respiratory function [6-9,16].

The effectiveness of the tidal volume challenge and end-expiratory occlusion tests

A 2022 study by Rui Shi et al. demonstrated the effectiveness of the TVC and EEO tests in predicting fluid responsiveness, particularly when compared to an >8% increase in cardiac index (CI) following the Trendelenburg maneuver [10]. The Trendelenburg maneuver is commonly used as a simple bedside test to assess fluid responsiveness, based on the premise that increasing venous return through head-down positioning will improve CO. However, it has known limitations, such as the potential to increase intra-abdominal pressure and exacerbate respiratory distress, particularly in patients with ARDS. The gold standard for fluid responsiveness testing typically involves a 10% increase in CI after administering a 100 mL colloid fluid bolus over one minute. While invasive methods like pulmonary artery catheterization remain the gold standard for measuring CO, non-invasive techniques such as transpulmonary thermodilution and pulse contour analysis are gaining popularity due to their comparable reliability and lower risk [11-13].

The objective of this study is to evaluate the effectiveness of the EEO and TVC tests in assessing fluid responsiveness in patients with severe TBI, septic shock, and ARDS, particularly those receiving low VT ventilation. While previous studies have primarily focused on general fluid responsiveness or patients not receiving low VT ventilation, this study specifically explores the challenges faced by these critically ill patients, expanding the understanding of how these tests can be applied in more complex clinical scenarios.

## Materials and methods

Study design

This study is a non-randomized prospective trial being conducted at Phramongkutklao Hospital. The aim is to enroll patients over the age of 20 who have been diagnosed with severe TBI, septic shock, and ARDS and who are currently receiving mechanical ventilation with a VT of 6 mL/kg of predicted body weight. The researcher is responsible for thoroughly explaining the research procedures to the patients or their relatives, informing them of any potential side effects, and obtaining informed consent. A non-randomized design was chosen due to the practical challenges of randomizing critically ill patients in intensive care settings, where patient characteristics and clinical conditions may necessitate tailored management. This design allows for the evaluation of fluid responsiveness tests in a real-world setting while maintaining patient safety and clinical relevance.

Study population

The study included all patients over 20 years of age who were diagnosed with severe TBI, septic shock, and ARDS and who were currently receiving mechanical ventilation with a VT of 6 mL/kg of predicted body weight. We excluded patients with abnormal heart rhythms (atrial or ventricular arrhythmias) and a heart rate exceeding 120 beats per minute because these conditions could significantly affect CO and the interpretation of fluid responsiveness measurements. Arrhythmias could lead to irregular blood flow, which might confound the results of tests like the EEO and TVC, which rely on stable cardiovascular parameters. A high heart rate could also limit the accuracy of these tests, as it might reduce the reliability of parameters such as stroke volume variation.

Patients with a history of pneumothorax were excluded because this condition can impair respiratory mechanics and complicate the interpretation of the EEO and TVC tests, which depend on stable and predictable ventilatory patterns. Additionally, patients or their relatives who declined participation were excluded to ensure informed consent was obtained and to avoid potential ethical issues.

Finally, patients with known allergies to any of the medications used in the study were excluded to avoid any adverse reactions that could interfere with the study's results and the patients' safety.

Hemodynamic monitoring

The radial arterial catheter and central venous catheter were connected to a pulse contour analysis CO monitoring system (FloTrac sensor®, HemoSphere® Advance monitor, Edwards Lifesciences). This setup allowed for continuous monitoring of key hemodynamic parameters, including blood pressure, CO, CI, PPV, and CVP.

Procedure

All patients were adequately sedated and paralyzed to facilitate respiratory control, following standard medical procedures. No fluid administration was performed before or during the testing procedures to avoid confounding factors affecting fluid responsiveness assessments.

Each patient underwent an EEO procedure for 15 seconds to record baseline CO measurements before and after the occlusion. This duration was selected based on previous studies, which demonstrated that a 15-second occlusion allows sufficient time for hemodynamic changes to stabilize and can accurately reflect fluid responsiveness in mechanically ventilated patients [8].

After a five-minute interval, a volume-controlled ventilation challenge was administered with TVs ranging from 6 mL/kg to 8 mL/kg of predicted body weight. This change in VT is expected to alter intrathoracic pressure and modify venous return, which can help assess the patient’s fluid responsiveness. The increase in VT mimics a transient positive fluid challenge, and the patient’s ability to respond to this challenge reflects their fluid responsiveness status.

Subsequently, PPV and CO measurements were taken. After another five-minute period, patients received a 100 mL colloid bolus over one minute, and further measurements were taken to assess changes in CO and fluid responsiveness.

The rationale for the cut-off values (5% for EEO and 3.5% for TVC) is that these thresholds were chosen based on previous studies and their validation as effective measures for distinguishing fluid responders from non-responders in mechanically ventilated patients. These values have been shown to optimize diagnostic performance, balancing sensitivity and specificity in detecting fluid responsiveness. The 5% threshold for EEO corresponds to the point where substantial hemodynamic changes are consistently observed, while the 3.5% threshold for TVC was selected based on studies that demonstrated its ability to predict fluid responsiveness with acceptable sensitivity and specificity.

The effect of changing VT on intrathoracic pressure is that an increase in VT from 6 mL/kg to 8 mL/kg induces a rise in intrathoracic pressure, which in turn impacts venous return and cardiac preload. This alteration can cause a temporary reduction in right ventricular filling, mimicking a fluid challenge. By assessing the patient’s response to this change, we can gauge the ability of the cardiovascular system to compensate for transient volume shifts, a key indicator of fluid responsiveness.

The study assumes that approximately 50% of patients will be fluid responders, based on the assumption of 50% fluid responders, a value often used in clinical practice and supported by previous research suggesting a roughly equal distribution of responders and non-responders in critically ill populations [9]. This assumption provides a basis for estimating the expected distribution of fluid responsiveness in this cohort, though further studies could refine this estimate for specific patient subgroups.

The necessity of paralyzing patients to obtain reliable readings

The necessity of paralyzing patients during the EEO test is an important consideration and may limit the applicability of the study to a wider patient population. The use of paralytics (neuromuscular blocking agents) is commonly employed to facilitate respiratory control during procedures like the EEO test, in order to minimize patient movement and ensure consistent, controlled ventilation. In mechanically ventilated patients, paralysis is often necessary to prevent involuntary movements that could disrupt the respiratory cycle, thereby affecting the precise measurement of variables such as CO and PPV.

In cases where strict control over ventilation is required to ensure accurate hemodynamic measurements, paralytics help eliminate confounding factors related to patient breathing efforts. This is particularly important when assessing dynamic parameters like fluid responsiveness, where even small changes in intrathoracic pressure can significantly affect the measurements.

Spontaneous breathing can introduce variability in CO measurements and other dynamic parameters, as respiratory efforts can influence intrathoracic pressure, venous return, and the accuracy of fluid responsiveness assessment. For instance, respiratory efforts during spontaneous breathing can cause fluctuations in intrathoracic pressure, which may interfere with measurements of PPV or stroke volume variation.

To improve the generalizability of the findings, future studies could explore alternatives to using paralytics for obtaining reliable fluid responsiveness readings. For example, techniques such as deep sedation without paralysis or adjusting ventilator settings to minimize spontaneous breathing could be considered. Additionally, new non-invasive hemodynamic monitoring methods or technologies for assessing fluid responsiveness without relying on paralytics may provide potential solutions for expanding the applicability of these tests.

Sample size

The required sample size, based on a 5% α risk and a 20% β risk, with an assumed 50% prevalence of preload responsiveness (as reported by Shi R. et al.), was calculated to be 45 participants [10].

Statistical analysis

The primary objective of this study was to evaluate whether changes in pulse pressure variation (△PPV6-8) during the volume-controlled ventilation challenge and alterations in CI during the end-expiratory occlusion (△CIEEO) could reliably determine preload responsiveness in patients positioned prone, with an area under the receiver operating characteristic curve (AUROC) of at least 0.9. The null hypothesis was set at 0.75, as an AUROC between 0.5 and 0.75 would suggest inadequate sensitivity and specificity for making meaningful conclusions.

Quantitative and categorical variables were expressed as mean ± SD and frequency (percentage), respectively. Data distribution was assessed using histograms to ensure normality. Statistical analyses were performed using appropriate tests based on the data type and distribution. For continuous variables, paired t-tests or Wilcoxon signed-rank tests were used to compare differences between baseline and maximal values, depending on the normality of the data. For categorical variables, chi-square tests or Fisher’s exact tests were employed to assess differences between responsive and non-responsive cases.

Receiver operating characteristic (ROC) curve analysis was used to assess the diagnostic performance of ΔPPV6-8 and △CIEEO, with AUROC values reported along with sensitivity, specificity, and their 95% confidence intervals. Additionally, p-values were calculated to assess interaction effects among different time points and between baseline and maximal values. A p-value of <0.05 was considered statistically significant for all analyses.

Ethics statement

The study was approved by the Ethics Committee of the Institutional Review Board, Royal Thai Army Medical Department, under opinion number IRBRTA 365/2567. It was conducted in accordance with the Declaration of Helsinki and the Foundation for Human Research Promotion in Thailand. Since this is a prospective study involving specific interventions, anonymized medical records, and other institutional clinical data presented in aggregate form that prevents participant identification, written informed consent was not required. However, verbal or implied consent was obtained from participants or their relatives, as per the guidelines set by the Council for International Organization of Medical Sciences (CIOMS) 2012 and the Good Clinical Practice (GCP) guidelines of the International Conference on Harmonization.

## Results

Patient characteristics

Among the 180 participants with severe TBI, septic shock, and ARDS, 63 (35%) were classified as fluid responders, and 117 (65%) were classified as fluid non-responders. The mean age in the response group was 64.29±13.4 years, compared to 59.18±19.65 years in the non-response group. Body mass index (BMI) was similar between the groups (22.23±2.84 vs. 22.18±2.91). The mean sequential organ failure assessment (SOFA) score was also comparable between the groups (6.15±0.7 vs. 6.4±1.13). Additionally, there was no significant difference in the acute physiology and chronic health evaluation (APACHE) II score (13.2±1.3 vs. 13.42±1.48), with a p-value of 0.541. The distribution of mechanisms of injury (blunt, penetrating, combined) was similar between the groups. The breakdown of injury mechanisms was as follows: blunt (162; 90% vs. 153; 85%) and penetrating (5; 2.78% vs. 11; 6.25%). Both groups had similar Glasgow Coma Score (GCS) scores (6.4±1.27 vs. 6.8±1.1). A higher proportion of the non-response group had hypertension (128; 71.1% vs. 69; 38.3%), with hypertension being the most common comorbid condition (Table 1). There were no statistically significant differences between the two groups for most variables, including age, BMI, SOFA, APACHE II, GCS, hematocrit, hemoglobin, norepinephrine dose, injury mechanisms, and underlying diseases. Hypertension showed a trend in the non-response group, but the difference was not statistically significant (p=0.16). Although systolic blood pressure was slightly higher in the response group, this difference was also not statistically significant (p=0.094), and diastolic blood pressure showed no significant difference between the groups (p=0.736). Heart rate and ejection fraction (EF) were similar across both groups, with no significant differences (p=0.91 and p=0.82, respectively). Despite some small differences (e.g., blood pressure and hypertension prevalence), the lack of statistical significance suggests that these variables may not be reliable indicators of fluid responsiveness in this population. Clinically, factors like blood pressure, comorbid hypertension, and injury severity could still influence treatment decisions, but their interpretation should be cautious in the absence of strong statistical evidence. The trends observed in variables such as hypertension and blood pressure may warrant further investigation, but their current lack of statistical significance suggests they are unlikely to play a major role in determining fluid responsiveness. Further analysis with larger sample sizes or additional variables could provide more insights.

**Table 1:** Demographic data for severe TBI, septic shock, and ARDS, categorized by fluid responders and non-responders The data are presented as follows: for continuous variables, values are expressed as mean ± SD, and for categorical variables, values are presented as N (%). The statistical tests used for comparisons are t-tests for continuous variables and chi-square tests for categorical variables. Statistical significance is considered at p<0.05, with values <0.001 indicated as p<0.001. The p-values in the tables are derived from the following statistical tests: t-test for comparing means of continuous variables between two groups (response vs. non-response) and chi-square test for comparing proportions or categorical variables between groups. The key variable being compared between the response and non-response groups. BMI: body mass index; SOFA: sequential organ failure assessment; APACHE2: acute physiology and chronic health evaluation 2; SBP: systolic blood pressure; DBP: diastolic blood pressure; GCS: Glasgow Coma Score; TBI: traumatic brain injury; ARDS: acute respiratory distress syndrome; EF: ejection fraction

	Response (n=63)	Non-response (n=117)	Test statistic	P-value
Male	102 (56.6%)	96 (53.3%)	χ²=0.02	0.887
Age (year)	64.29±13.4	59.18±19.65	t=0.61	0.548
BMI (kg/m^2^)	22.23±2.84	22.18±2.91	t=0.58	0.57
SOFA	6.15±0.7	6.4±1.13	t=-0.49	0.615
APACHE2	13.2±1.3	13.42±1.48	t=-0.63	0.541
Hematocrit at admission (%)	30.42±6.23	30.54±6.62	t=-0.37	0.717
Hemoglobin at admission (g/L)	7.1±1.72	7.13±1.48	t=-0.07	0.712
Dose norepinephrine at admission	0.14±0.07	0.13±0.08	t=1.17	0.242
Mechanism of injury				
Blunt	162 (90%)	153 (85%)	χ²=1.25	1
Penetrating	5 (2.78%)	11 (6.25%)	χ²=1.57	0.231
Combined blunt and penetrating	5 (2.78%)	11 (6.25%)	χ²=1.57	0.231
Accidental	171 (95%)	162 (90%)	χ²=1.14	1
Self-inflicted	5 (2.78%)	11 (6.25%)	χ²=1.57	0.231
Assault	5 (2.78%)	11 (6.25%)	χ²=1.57	0.231
GCS at admission	6.4±1.27	6.8±1.1	t=-1.20	0.85
Pupillary function				
Equal reaction to light in both eyes	166 (92.5%)	166 (92.5%)	χ²=0.00	1
Unequal reaction to light in both eyes	9 (5.5%)	11 (6.25%)	χ²=0.41	0.526
Not react to light in both eyes	5 (2.78%)	11 (6.25%)	χ²=1.46	0.231
Marshall score on admission				
Diffuse injury 1	9 (5.5%)	11 (6.25%)	χ²=0.08	0.526
Diffuse injury 2	5 (2.78%)	11 (6.25%)	χ²=1.43	0.231
Diffuse injury 3	84 (47.2%)	101 (56.3%)	χ²=0.56	0.480
Diffuse injury 4	45 (25%)	33 (18.75%)	χ²=0.10	0.751
Diffuse injury 5	9 (5.5%)	11 (6.25%)	χ²=0.08	0.526
Diffuse injury 6	9 (5.5%)	11 (6.25%)	χ²=0.08	0.526
Surgery performed	52 (29.4%)	90 (50%)	χ²=2.27	0.39
Underlying disease				
Hypertension	128 (71.1%)	69 (38.3%)	χ²=2.14	0.16
Diabetes mellitus	25 (13.89%)	27 (15%)	χ²=0.01	0.948
Dyslipidemia	31 (17.6%)	22 (12.5%)	χ²=1.66	1
Coronary artery disease	21 (11.8%)	22 (12.5%)	χ²=0.01	1
Previous cerebrovascular disease	21 (11.8%)	22 (12.5%)	χ²=0.01	1
SBP (mmHg)	95.52±8.61	90.12±4.81	t=1.67	0.094
DBP (mmHg)	51.25±6.72	50.42±4.27	t=0.33	0.736
Heart rate (min)	76±18.68	74.17±19.57	t=0.12	0.91
EF (%)	55±7.52	53±8.65	t=0.76	0.82
Weight (kg)	63.4±12.62	64.71±8.84	t=-0.14	0.9
Height (cm)	165±10.95	158.23±6.98	t=1.15	0.248

End-expiratory occlusion test

An increase in CI greater than 5% effectively distinguishes fluid responders from non-responders (p=0.012), demonstrating a sensitivity of 100%, specificity of 92.3%, and an overall accuracy of 95%. This 5% threshold was likely determined based on previous studies, which identified similar cut-offs for fluid responsiveness, providing a robust foundation for its use [4,5]. Further analysis revealed that a 4.6% increase in CI from baseline serves as the optimal cut-off for identifying fluid responders, with a Youden index of 0.923 (Tables 2, 3). This 4.6% threshold maximizes the balance between sensitivity and specificity, offering the best diagnostic accuracy.

**Table 2:** Mean differences in hemodynamic variables for severe TBI, septic shock, and ARDS between the fluid responder and non-responder groups ^*^Statistical significance at p<0.05. The data are presented as follows: for continuous variables, values are expressed as mean ± SD, and for categorical variables, values are presented as N (%). The statistical tests used for comparisons are t-tests for continuous variables and chi-square tests for categorical variables. The Mean difference (95% CI) is shown along with the p-value. Statistical significance is considered at p<0.05, with values <0.001 indicated as p<0.001. CO: cardiac output; CI: cardiac index; PPV: pulse pressure variation; FL: fluid loading; EEO: end-expiratory occlusion; TBI: traumatic brain injury; ARDS: acute respiratory distress syndrome

	Response (n=63)	Non-response (n=117)	Mean difference (95%CI)	Test statistic	P-value
CO1	4.1±0.64	4.13±1.02	-0.03 (-0.92, 0.86)	t=-0.07	0.942
CI1	2.41±0.28	2.46±0.61	-0.04 (-0.56, 0.48)	t=-0.18	0.865
CO EEO	4.5±0.67	4.25±1.04	0.25 (-0.67, 1.16)	t=0.57	0.576
CI EEO	2.65±0.28	2.53±0.62	0.12 (-0.41, 0.65)	t=0.47	0.647
△CIEEO%	9.94±5.15	3.02±0.97	6.91 (2.15, 11.68)	t=2.57	0.012*
PPV6	8.43±5.13	7.62±1.76	0.81 (-3.94, 5.57)	t=0.39	0.697
PPV8	12.86±6.41	9.46±2.3	3.4 (-2.56, 9.35)	t=1.23	0.219
△PPV6-8	4.43±1.99	1.85±1.21	2.58 (1.09, 4.08)	t=3.47	0.002*
CO2	4±0.59	4.09±1.02	-0.09 (-0.98, 0.79)	t=-0.22	0.829
CI2	2.36±0.29	2.44±0.63	-0.08 (-0.61, 0.46)	t=-0.26	0.767
CO FL	4.97±0.97	4.45±1.07	0.52 (-0.5, 1.54)	t=1.01	0.301
CI FL	2.93±0.54	2.65±0.66	0.28 (-0.33, 0.9)	t=0.96	0.35

**Table 3:** Statistical results of various cut-off values to differentiate between fluid responders and non-responders, identifying the optimal threshold for discerning fluid responders in severe TBI, septic shock, and ARDS ^***^Statistical significance at the specific cutoff point for △CI% and △PPV. The table presents the sensitivity, specificity, PPV, NPV, and accuracy for various cutoff values of the variables △CI% and △PPV. Test Statistic: χ² (chi-square value) for comparisons of proportions across different cut-offs. P-value, statistical significance value derived from the chi-square test. A p-value of <0.05 indicates statistical significance, with values marked as p<0.001 indicating highly significant results. △CI: difference in the cardiac index; △PPV: difference in pulse pressure variation; PPV: positive predictive value; NPV: negative predictive value; TBI: traumatic brain injury; ARDS: acute respiratory distress syndrome

Variable(s)	Cut off	Sensitivity	Specificity	PPV	NPV	Accuracy	Test statistic (χ²)	P-value
△CI%	2.15	100.0%	7.7%	36.8%	100.0%	40.0%	68.4	<0.001
	2.50	100.0%	30.8%	43.8%	100.0%	55.0%	40.1	<0.001
	2.67	100.0%	46.2%	50.0%	100.0%	65.0%	31.2	<0.001
	2.86	100.0%	53.8%	53.8%	100.0%	70.0%	25.6	<0.001
	2.94	100.0%	61.5%	58.3%	100.0%	75.0%	22.3	<0.001
	3.14	100.0%	69.2%	63.6%	100.0%	80.0%	19.5	<0.001
	3.33	100.0%	76.9%	70.0%	100.0%	85.0%	16.7	<0.001
	3.71	100.0%	84.6%	77.8%	100.0%	90.0%	14.3	<0.001
***	4.60	100.0%	92.3%	87.5%	100.0%	95.0%	13.2	<0.001
	5.20	85.7%	92.3%	85.7%	92.3%	90.0%	10.8	<0.001
	5.41	71.4%	92.3%	83.3%	85.7%	85.0%	9.3	<0.001
	5.63	57.1%	92.3%	80.0%	80.0%	80.0%	7.1	<0.001
	6.91	57.1%	100.0%	100.0%	81.3%	85.0%	5.0	<0.001
	14.19	28.6%	100.0%	100.0%	72.2%	75.0%	3.5	<0.001
	16.35	14.3%	100.0%	100.0%	68.4%	70.0%	2.1	<0.001
△ PPV	1	100.0%	15.4%	38.9%	100.0%	45.0%	68.4	<0.001
	2	100.0%	38.5%	46.7%	100.0%	60.0%	47.5	<0.001
***	3	85.7%	69.2%	60.0%	90.0%	75.0%	32.2	<0.001
	4	57.1%	92.3%	80.0%	80.0%	80.0%	28.9	<0.001
	5	42.9%	100.0%	100.0%	76.5%	80.0%	24.4	<0.001
	6	28.6%	100.0%	100.0%	72.2%	75.0%	20.1	<0.001

While the 5% threshold is effective for distinguishing responders, the 4.6% cut-off, derived from further analysis, is considered optimal [7]. The 5% threshold is valuable for general clinical practice, providing high sensitivity and specificity [9]. However, the 4.6% threshold offers a more precise diagnostic criterion with the highest Youden index, which represents the best overall test performance by maximizing both sensitivity and specificity. Depending on the clinical context, either threshold may be used, but the 4.6% threshold is recommended for its optimal diagnostic accuracy.

Tidal volume challenge test

An increase in PPV greater than 3.5% significantly differentiates fluid responders from non-responders (p=0.002), with a sensitivity of 57.1%, specificity of 92.3%, and an overall accuracy of 80%. While this threshold is useful, the relatively low sensitivity suggests that the test may miss a significant portion of fluid responders. Further analysis reveals that the optimal threshold for identifying fluid responders is a 3.0% increase in PPV from baseline, corresponding to a Youden index of 0.549 (Tables 2, 3). Although the 3.0% threshold improves diagnostic performance, the sensitivity remains relatively low. In critical care settings, where the early identification of fluid responders is crucial for improving patient outcomes, this limitation could be significant. Missing fluid responders due to low sensitivity may result in suboptimal management, potentially affecting patient recovery. Thus, while the TVC test may be valuable in certain contexts, its limited sensitivity should be considered when using it to guide treatment decisions.

End-expiratory occlusionand tidal volume challenge

In the comparison between the EEO and TVC tests, both demonstrated statistically significant assessments of fluid responsiveness. The AUROC for EEO is 0.978 (p=0.001), while for TVC, it is 0.885 (p=0.007). Regarding reliability, EEO shows greater consistency than TVC, with kappa values of 0.915 and 0.637, respectively (Table 4).

**Table 4:** Comparison of the EEO and TVC tests in assessing fluid responsiveness in severe TBI, septic shock, and ARDS ^*^Statistical significance at p<0.05. The table presents the AUC, 95% CI, paired AUC, and Kappa values. The Z-value was used for comparing the AUC values to a reference (0.5). A Z-value is computed to assess if the AUC is significantly different from 0.5, which is equivalent to random guessing. Cohen's Kappa statistic is used to assess the degree of agreement between the paired results. A higher Kappa value indicates stronger agreement. P-values below 0.05 (p<0.05) are considered statistically significant. △CI: Difference in cardiac index; △PPV: Difference in pulse pressure variation: EEO: end-expiratory occlusion; TVC: tidal volume challenge; TBI: traumatic brain injury; ARDS: acute respiratory distress syndrome; AUC: area under the curve; TBI: traumatic brain injury; ARDS: acute respiratory distress syndrome

Variable(s)	AUC	95% CI		Test statistic (Z)	P-value	Paired AUC	Kappa
		Lower bound	Upper bound			P-value	
△CI%	0.978	0.894	1	3.09	0.001*	0.213	0.915
△PPV	0.885	0.715	1	2.68	0.007*		0.637

## Discussion

End-expiratory occlusion and tidal volume challenge

The findings of our study indicate that the EEO test demonstrates a higher degree of reliability in differentiating between fluid-responsive and non-responsive states compared to the TVC test. This contrasts with a previous study by Rui Shi, in which both methods were found to be equally effective [10]. The observed discrepancy may be due to differences in the standards used to classify fluid responsiveness. In Rui Shi's study, the Trendelenburg test was used as the reference standard, whereas our study employed the administration of 100 mL of colloid fluid bolus as the reference standard for assessing fluid responsiveness. Additionally, our study specifically focused on patients with severe TBI, septic shock, and ARDS, while Rui Shi's study assessed fluid responsiveness across a broader range of patient populations [13].

A recent 2021 study by Alvarado Sanchez JL et al. demonstrated that, among patients ventilated with low TVs, both the TVC and EEO methods outperformed other tests in determining fluid responsiveness [14]. However, it was noted that the methods used to classify patients as fluid responders or non-responders after final fluid loading significantly influenced the reliability of PPV and, consequently, the TVC test [13].

The tidal volume challenge test**

The TVC test evaluates fluid responsiveness by temporarily increasing the VT during mechanical ventilation and observing changes in stroke volume or CO. By amplifying the respiratory variations in stroke volume through a higher VT, this test helps assess the patient's capacity to respond to fluid administration.

Advantages in severe traumatic brain injury with septic shock and acute respiratory distress syndrome

Advantages in severe TBI, septic shock, and ARDS include real-time monitoring. The TVC test provides immediate feedback on a patient's hemodynamic status, enabling clinicians to make rapid decisions about fluid administration. It is particularly valuable in time-sensitive situations. For mechanically ventilated patients with ARDS, the TVC test can be synchronized with the ventilator, avoiding the need for additional invasive monitoring or complex setups. This method leverages the patient’s existing ventilation to assess fluid responsiveness. Additionally, the TVC test is sensitive to respiratory changes, making it especially useful in ARDS patients, as it directly evaluates the stroke volume's response to ventilatory variations, offering valuable insight into how the patient might respond to fluids amidst pulmonary compromise.

Potential disadvantages

Considerations and risks in TBI, septic shock, and ARDS include the impact on respiratory mechanics. In ARDS patients, increasing the VT can worsen ventilatory issues, potentially causing barotrauma or volutrauma, which can further complicate respiratory management. Therefore, it is crucial to ensure that VT remains within safe limits to avoid exacerbating pulmonary injury in these patients.

Intracranial pressure

ICP in TBI, similar to the EEO test, the TVC test may cause transient changes in intrathoracic pressure, potentially affecting ICP in patients with TBI. Therefore, close monitoring of ICP during the test is crucial to minimize the risk of worsening neurological injury.

Sepsis and myocardial dysfunction

In patients with severe TBI, septic shock, and ARDS, fluid resuscitation is critical for management. However, assessing fluid responsiveness in these patients can be challenging due to the complex pathophysiology at play. Myocardial dysfunction in septic shock can impair the heart’s ability to increase stroke volume in response to changes in intrathoracic pressure, leading to false-negative results in fluid responsiveness tests, and potentially suboptimal resuscitation.

The EEO and TVC tests are valuable methods for assessing fluid responsiveness. The EEO test evaluates the cardiovascular response to changes in intrathoracic pressure during the mechanical ventilation cycle, while the TVC test assesses the heart’s ability to increase stroke volume in response to ventilatory adjustments (i.e., changes in VT). These tests provide complementary insights into how the heart responds to dynamic changes in intrathoracic pressure and stroke volume.

Cardiac performance and fluid responsiveness

Cardiac performance, especially the estimated EF, is crucial when interpreting the results of the EEO and TVC tests. A reduced EF, which reflects impaired myocardial contractility commonly seen in septic shock and ARDS, can limit the heart’s capacity to appropriately increase stroke volume in response to intrathoracic pressure changes (EEO) or ventilatory adjustments (TVC). Therefore, it is essential to account for the patient’s underlying cardiac function when interpreting fluid responsiveness.

In cases of myocardial dysfunction, even if the EEO and TVC tests show no significant changes in stroke volume in response to ventilatory adjustments, this may not necessarily indicate that the patient is not fluid-responsive. Instead, the lack of response could be due to impaired contractility, limiting the heart's ability to increase stroke volume. Relying solely on these tests without considering cardiac performance could lead to a misinterpretation of fluid responsiveness, resulting in inadequate fluid therapy.

Thus, integrating the EEO and TVC tests with continuous monitoring of cardiac performance, such as EF, provides a more reliable and individualized approach to fluid management. By understanding how myocardial dysfunction impacts the heart’s response to changes in intrathoracic pressure and stroke volume, clinicians can make more informed decisions regarding fluid therapy. This approach ensures optimal resuscitation strategies and reduces the risk of over- or under-resuscitation in critically ill patients with complex conditions like severe TBI, septic shock, and ARDS.

The correlation between the EEO and TVC tests with cardiac performance (including EF) is essential for accurate assessment of fluid responsiveness. This comprehensive approach enables clinicians to tailor fluid therapy more effectively, ultimately improving outcomes in these high-risk patients [13,16].

Clinical integration considerations

Clinical integration considerations include the need for close monitoring of ICP. Both the EEO and TVC tests involve changes in intrathoracic pressure, which can affect ICP. Continuous monitoring of ICP is essential, especially in TBI patients, to prevent exacerbation of brain injury [13].

Fluid overload risk

Consider the risk of fluid overload while both the EEO and TVC tests are useful for assessing fluid responsiveness; however, their results should be interpreted alongside other clinical parameters, particularly in ARDS patients. This is crucial to avoid fluid overload, which can exacerbate pulmonary edema and impair oxygenation [14].

Multimodal approach

These tests should be integrated into a comprehensive approach to assess fluid responsiveness, which includes monitoring hemodynamic parameters (e.g., mean arterial pressure, lactate levels, and urine output), respiratory function (e.g., oxygenation and compliance), and clinical status (e.g., mental status and tissue perfusion) [14].

Limitations

In line with our findings, the EEO test demonstrated higher reliability compared to the TVC test. One reason for this disparity may be related to the limitations in the equipment used to measure minor differences in VT, as the TVC test involves small changes in VT (as little as 2 mL). Such a minimal change is likely to produce a less significant impact on intrathoracic pressure, potentially limiting the effectiveness of the test in capturing meaningful variations in fluid responsiveness [10].

Furthermore, the dynamic changes in intrathoracic pressure associated with ARDS may be more pronounced than in non-ARDS patients. This is due to the pathophysiological alterations in lung compliance and the elevated pressure required to maintain adequate oxygenation in ARDS. These factors could influence the reliability of established thresholds for the TVC test, making it less accurate or more variable in ARDS patients [13].

Additionally, the TVC test might still have potential value in assessing fluid responsiveness, as suggested by previous studies, including the work by Rui Shi. In their research, the TVC test was correlated with fluid responsiveness, particularly when patients responded positively to fluid boluses. However, the effectiveness of the TVC test is still under investigation, and further studies with larger sample sizes are essential to conclusively establish its role in evaluating fluid responsiveness across diverse patient populations [14-16].

The effects of medications are an essential confounding factor when interpreting fluid responsiveness tests, particularly in critically ill patients with TBI, septic shock, and ARDS. Medications like vasopressors, inotropes, sedatives, diuretics, and corticosteroids can significantly influence the results of the EEO and TVC tests by altering cardiovascular dynamics, respiratory mechanics, or fluid status. These factors should be carefully considered when applying these tests in clinical practice, and ideally, future studies would integrate an analysis of medication effects on fluid responsiveness testing to provide a more nuanced understanding of their influence [5].

Vasopressors could alter the relationship between changes in intrathoracic pressure and stroke volume, potentially leading to misleading results in both the EEO and TVC tests. Vasopressors may artificially improve stroke volume or blood pressure, masking the true fluid responsiveness or creating false positives [5,6].

Inotropes, these medications aim to increase myocardial contractility, which could alter how the heart responds to changes in preload (i.e., changes in intrathoracic pressure from the EEO or TVC tests). Inotropes could lead to exaggerated responses in fluid responsiveness tests, potentially overstating a patient’s capacity to handle fluid resuscitation [5,6].

Medications like propofol, benzodiazepines, and opioids are commonly administered in patients with TBI or ARDS for sedation or analgesia. These medications can alter respiratory mechanics and affect sympathetic tone. Sedation may lead to reduced respiratory drive or ventilator dependence, which could reduce the effectiveness or sensitivity of the TVC test. Since the TVC test relies on respiratory variations in stroke volume, any dampening of the ventilatory response might lead to inaccurate assessments of fluid responsiveness. Sedatives and analgesics can influence the autonomic nervous system, potentially leading to reduced vasomotor tone and changes in heart rate and blood pressure, thus affecting fluid responsiveness [2-4].

Corticosteroids, often used in ARDS management, especially in refractory cases, can alter fluid balance and cardiovascular function by increasing fluid retention and affecting vascular reactivity. Corticosteroids promote sodium retention and fluid retention, which could influence the EEO test by artificially increasing preload and altering the patient’s fluid status. This might reduce the sensitivity of the test in predicting true fluid responsiveness. Corticosteroids can affect vascular smooth muscle function and may also modify the response to changes in intrathoracic pressure observed during the TVC test, potentially masking the true fluid responsiveness of the patient [2,3].

Diuretics (e.g., furosemide) are frequently used in patients with ARDS or fluid overload. Their effects on fluid responsiveness testing can be significantly reduced preload and complicated interpretation of fluid status. By reducing circulating volume, diuretics might decrease the preload, which is an essential determinant of fluid responsiveness. In patients on diuretics, both the EEO and TVC tests could show diminished responses due to a reduced ability to expand the intravascular volume. Diuretics can complicate the interpretation of fluid responsiveness because they alter baseline volume status. A patient on diuretics may appear less fluid-responsive even though they might require fluid resuscitation due to ongoing hypovolemia or electrolyte imbalances [2-4].

Patients with septic shock are often treated with antibiotics, which can alter cardiovascular dynamics in a variety of ways. Some antibiotics, particularly those that cause significant hypotension (e.g., vancomycin or piperacillin-tazobactam), might impact the reliability of fluid responsiveness tests. If a patient’s blood pressure drops in response to antibiotic therapy, the cardiovascular response to fluid challenges may be blunted, leading to misleading test results. Other supportive medications, like antihypertensives, may also interfere with fluid responsiveness assessments by modifying baseline hemodynamic status [13,14].

Certain medications, especially those that modulate the autonomic nervous system, such as beta-blockers (e.g., propranolol) or alpha-2 agonists (e.g., clonidine), could influence the body’s response to changes in intrathoracic pressure or ventilatory adjustments. Beta-blockers, for instance, might blunt the heart rate response during fluid responsiveness tests, leading to diminished changes in stroke volume. Similarly, medications affecting peripheral vascular resistance could alter how the body compensates for changes in circulating volume and stroke volume, thereby interfering with the results of the TVC and EEO tests [16].

Pharmacodynamic interactions between medications could also play a role in modifying fluid responsiveness. For example, the combined use of vasopressors and inotropes may have synergistic effects that alter how the body responds to fluid challenges. These interactions can lead to unpredictable results in fluid responsiveness testing, especially in critically ill patients with complex comorbidities [16].

By incorporating a more detailed examination of medication effects, clinicians can better interpret the results of these tests, improving fluid management strategies for critically ill patients.

## Conclusions

In patients with severe TBI, septic shock, and ARDS undergoing mechanical ventilation with low tidal volumes, both the EEO and TVC tests have been shown to be effective in assessing fluid responsiveness. However, the EEO test demonstrates greater reliability compared to the TVC test. These findings suggest that, in this specific patient population, the EEO test may be the preferred method for assessing fluid responsiveness due to its superior reliability. Nevertheless, the TVC test still offers value, particularly in cases where EEO may not be available or suitable, and it may still have a role in clinical practice as a complementary tool.

Furthermore, the study highlights the need for careful monitoring of patients with severe TBI, septic shock, and ARDS, as fluid responsiveness significantly influences treatment decisions and recovery trajectories. While both tests provide valuable insights, understanding their limitations and advantages in different clinical contexts is essential. Future research should focus on refining these techniques and exploring their application in larger, more diverse patient populations to confirm the generalizability of these findings and potentially enhance patient management across various critical care settings.

## References

[1] Mikkelsen ME, Shah CV, Meyer NJ (2013). The epidemiology of acute respiratory distress syndrome in patients presenting to the emergency department with severe sepsis. Shock.

[2] Qadir N, Sahetya S, Munshi L (2024). An update on management of adult patients with acute respiratory distress syndrome: an official American Thoracic Society clinical practice guideline. Am J Respir Crit Care Med.

[3] Gorman EA, O’Kane CM, McAuley DF (2022). Acute respiratory distress syndrome in adults: diagnosis, outcomes, long-term sequelae, and management. Lancet.

[4] Shi R, Monnet X, Teboul JL (2020). Parameters of fluid responsiveness. Curr Opin Crit Care.

[5] Marik PE, Monnet X, Teboul JL (2011). Hemodynamic parameters to guide fluid therapy. Ann Intensive Care.

[6] De Backer D, Aissaoui N, Cecconi M (2022). How can assessing hemodynamics help to assess volume status?. Intensive Care Med.

[7] Gavelli F, Shi R, Teboul JL, Azzolina D, Monnet X (2020). The end-expiratory occlusion test for detecting preload responsiveness: a systematic review and meta-analysis. Ann Intensive Care.

[8] Gavelli F, Teboul JL, Monnet X (2019). The end-expiratory occlusion test: please, let me hold your breath!. Crit Care.

[9] Messina A, Montagnini C, Cammarota G (2020). Assessment of fluid responsiveness in prone neurosurgical patients undergoing protective ventilation: role of dynamic indices, tidal volume challenge, and end-expiratory occlusion test. Anesth Analg.

[10] Shi R, Ayed S, Moretto F (2022). Tidal volume challenge to predict preload responsiveness in patients with acute respiratory distress syndrome under prone position. Crit Care.

[11] McGee WT, Horswell JL, Calderon J, Janvier G, Van Severen T, Van den Berghe G, Kozikowski L (2007). Validation of a continuous, arterial pressure-based cardiac output measurement: a multicenter, prospective clinical trial. Crit Care.

[12] Arya VK, Al-Moustadi W, Dutta V (2022). Cardiac output monitoring - invasive and noninvasive. Curr Opin Crit Care.

[13] Hofer CK, Senn A, Weibel L, Zollinger A (2008). Assessment of stroke volume variation for prediction of fluid responsiveness using the modified FloTrac and PiCCOplus system. Crit Care.

[14] Alvarado Sánchez JI, Caicedo Ruiz JD, Diaztagle Fernández JJ, Amaya Zuñiga WF, Ospina-Tascón GA, Cruz Martínez LE (2021). Predictors of fluid responsiveness in critically ill patients mechanically ventilated at low tidal volumes: systematic review and meta-analysis. Ann Intensive Care.

[15] Zivkovic AR, Kjaev A, Schönenberger S, Krieg SM, Weigand MA, Neumann JO (2024). Feasibility of fluid responsiveness assessment in patients at risk for increased intracranial pressure. J Clin Med.

[16] Selvam V, Shende D, Anand RK, Kashyap L, Ray BR (2023). End-expiratory occlusion test and mini-fluid challenge test for predicting fluid responsiveness in acute circulatory failure. J Emerg Trauma Shock.

